# Robust fingerprinting of genomic databases

**DOI:** 10.1093/bioinformatics/btac243

**Published:** 2022-06-27

**Authors:** Tianxi Ji, Erman Ayday, Emre Yilmaz, Pan Li

**Affiliations:** Department of Electrical, Computer, and System Engineering, Case Western Reserve University, Cleveland, OH 44106, USA; Department of Computer and Data Sciences, Case Western Reserve University, Cleveland, OH 44106, USA; Department of Computer Science and Engineering Technology, University of Houston-Downtown, Houston, TX 77002, USA; Department of Electrical, Computer, and System Engineering, Case Western Reserve University, Cleveland, OH 44106, USA

## Abstract

**Motivation:**

Database fingerprinting has been widely used to discourage unauthorized redistribution of data by providing means to identify the source of data leakages. However, there is no fingerprinting scheme aiming at achieving liability guarantees when sharing genomic databases. Thus, we are motivated to fill in this gap by devising a vanilla fingerprinting scheme specifically for genomic databases. Moreover, since malicious genomic database recipients may compromise the embedded fingerprint (distort the steganographic marks, i.e. the embedded fingerprint bit-string) by launching effective correlation attacks, which leverage the intrinsic correlations among genomic data (e.g. Mendel’s law and linkage disequilibrium), we also augment the vanilla scheme by developing mitigation techniques to achieve robust fingerprinting of genomic databases against correlation attacks.

**Results:**

Via experiments using a real-world genomic database, we first show that correlation attacks against fingerprinting schemes for genomic databases are very powerful. In particular, the correlation attacks can distort more than half of the fingerprint bits by causing a small utility loss (e.g. database accuracy and consistency of SNP–phenotype associations measured via *P*-values). Next, we experimentally show that the correlation attacks can be effectively mitigated by our proposed mitigation techniques. We validate that the attacker can hardly compromise a large portion of the fingerprint bits even if it pays a higher cost in terms of degradation of the database utility. For example, with around 24% loss in accuracy and 20% loss in the consistency of SNP–phenotype associations, the attacker can only distort about 30% fingerprint bits, which is insufficient for it to avoid being accused. We also show that the proposed mitigation techniques also preserve the utility of the shared genomic databases, e.g. the mitigation techniques only lead to around 3% loss in accuracy.

**Availability and implementation:**

https://github.com/xiutianxi/robust-genomic-fp-github.

## 1 Introduction

Genomic database sharing is critical in modern biomedical research, clinical practice and customized healthcare. However, it is generally not viable due to the copyright and intellectual property concerns from the database owners. In other words, the requirements of copyright protection and anti-piracy may prevent genomic data holders from sharing their data, which may hinder the progress of cooperative scientific research.

Digital fingerprinting is a technology that allows to claim copyright, deter illegal redistribution and identify the source of data breaches (i.e. the guilty party who is responsible for the leakage) by embedding a unique mark into each shared copy of a digital object. Although the most prominent usage of fingerprinting is for multimedia (Cox *et al.*, 1997, 2002; Johnson *et al.*, 2001), fingerprinting techniques for databases have also been developed (Guo *et al.*, 2006; Lafaye *et al.*, 2008; Li *et al.*, 2005; Liu *et al.*, 2004). These techniques change database entries at different positions when sharing a database copy with a new service provider (SP). If the SP shares its received copy without authorization, the database owner can use the inserted fingerprints to hold the guilty SP responsible.

### 1.1 Challenges in genomic database fingerprinting

Existing fingerprinting schemes for databases have been developed to embed fingerprints in continuous numerical entries (floating points) in relational databases, e.g. Li *et al.* (2005), Guo *et al.* (2006) and Li *et al.* (2003). Whereas, fingerprinting discrete (or categorical) values is more difficult, as the number of possible values (or instances) for a data point is much fewer. Hence, in such databases, a small change in data points (as a fingerprint) can significantly affect the utility. Fingerprinting becomes even more challenging when it comes to a genomic databases, which contain even fewer values, e.g. four instances (A, G, C and T) when considering nucleobases, and three instances (0, 1 and 2) when considering number of minor alleles for each single-nucleotide polymorphism (SNP) (Section 3 gives more details about the type of genomic data considered in this work). In real world, leaked genomic databases often end up being sold or publicly shared on the internet (McGee and Ross, 2016). Once that happens, the genomic database owner wants to find out the traitors who should be responsible for the data leakage by extracting their fingerprints in the leaked databases. Thus, in this article, we first propose a vanilla genomic database fingerprinting scheme by (i) taking into account of the abundant attributes of genomic sequence, and (ii) extending the state-of-the-art database fingerprinting scheme (Li *et al.*, 2005). Our vanilla scheme is more robust against common attacks targeted on database fingerprinting schemes [e.g. random bit flipping attack and subset attack (Agrawal *et al.*, 2003; Li *et al.*, 2003)] than previously developed fingerprinting schemes for generic relational databases, such as Li *et al.* (2005), Guo *et al.* (2006) and Liu *et al.* (2004), because we can insert denser fingerprint for each selected genomic data by introducing a new parameter, which controls the percentage of fingerprinted entries for selected rows (see Section 4.1). Whereas, Li *et al.* (2005) only fingerprints one attribute for all selected rows. Compared with Li *et al.* (2005), we also assign higher confidence score during fingerprint extraction considering that genomic databases usually contain more attributes than generic databases (see Section 4.2).

In addition, existing fingerprinting schemes for databases do not consider various inherent correlations between the data records in a database. In our previous work (Ji *et al.*, 2021a), we have shown that a malicious party having a fingerprinted copy of a database can detect and distort the embedded fingerprints using its knowledge about the correlations in the data entries. Genomic databases contain even richer row- and column-wise correlations due to the biological characteristics. In particular, the row-wise correlations arise from (i) the Mendel’s law, and (ii) similarities of genomes among family members. The column-wise correlations are the pair-wise correlation between genomic data points at different locations [e.g. linkage disequilibrium (Naveed *et al.*, 2015)]. In this article, we use ${\text{Atk}}_{\text{row}}(S)$ and ${\text{Atk}}_{\text{col}}(J)$ to represent the correlation attacks using the row- and column-wise correlations, respectively, where S and J denote the corresponding correlation model and they are assumed to be publicly known (in Section 3.2, we describe these two attacks in detail). In Section 6, we consider a real-world genomic database and show that by launching ${\text{Atk}}_{\text{row}}(S)$ and ${\text{Atk}}_{\text{col}}(J)$ in sequence, a malicious SP can easily compromise more than half of the bits in a fingerprint string at the cost of only changing about 5% of the entries in the genomic databases. As a result, we also need to make the proposed vanilla genomic database fingerprinting scheme be robust against the correlation attacks in order to lay a solid foundation for genomic data sharing.

### 1.2 Our solution

In this work, to address the unique challenges of robust fingerprinting of genomic databases, i.e. mitigating ${\text{Atk}}_{\text{row}}(S)$ and ${\text{Atk}}_{\text{col}}(J)$, we develop mitigation techniques for each of them, i.e. ${\text{Mtg}}_{\text{row}}(S)$ and ${\text{Mtg}}_{\text{col}}(J)$. These techniques utilize the correlations among genomic data, i.e. Mendel’s law, S, and J, and they work as post-processing steps for our developed vanilla scheme. Besides, they only modify non-fingerprinted entries in the genomic databases. Thus, they do not reduce the robustness of the vanilla scheme. Note that the proposed robust genomic database fingerprinting scheme in this article is not just a simple application of our previous work (Ji *et al.*, 2021a) for genomic databases, because the correlation models considered in this article are different compared to the generic models we have (Ji *et al.*, 2021a), and thus they require new mitigation techniques to make the fingerprinted genomic databases match the Mendel’s law and genome-specific correlations. In Table 1, we summarize the differences between the proposed robust genomic database fingerprinting scheme and previous schemes.

**Table 1.** Differences between the robust genomic database fingerprinting and the previous schemes

Properties	Li *et al.* (2005)	Ji *et al.* (2021a)	This article
Flexible density in marked attributes	✗	✗	✓
Higher confidence in extraction	✗	✗	✓
Genome-specific correlation	✗	✗	✓

*Note*: ✓ indicates the scheme has a certain property, and ✗ indicates the opposite.

In particular, ${\text{Mtg}}_{\text{row}}(S)$ is composed of two phases. First, it checks all fingerprinted genomic data-tuples of family members. If a tuple violates Mendel’s law, the database owner changes the non-fingerprinted entries in this tuple to make it compliant with the Mendel’s law. Second, it checks all family sets in the genomic database, calculates the empirical correlations among family members after the vanilla fingerprinting, and changes the non-fingerprinted entries in each family set to push the empirical correlations close to the publicly known model (S) by solving a distance minimization problem. Note that the second phase of ${\text{Mtg}}_{\text{row}}(S)$ is different with the row-wise mitigation developed in our previous work (Ji *et al.*, 2021a), because the second phase is able to perfectly defend against ${\text{Atk}}_{\text{row}}(S)$ if the objective function of the distance minimization problem reaches to 0. Whereas, our previous row-wise mitigation technique (Ji *et al.*, 2021a) (formulated as a set function maximization problem) can only mislead the malicious SP when launching the row-wise correlation attack. More details are deferred to Section 5.1.

${\text{Mtg}}_{\text{col}}(J)$ considers all attributes (columns) of the genomic databases, obtains the empirical marginal distributions after the vanilla fingerprinting, and changes non-fingerprinted entries in each attribute to make the empirical marginal distributions resemble the marginal distributions obtained by marginalizing the joint distributions provided in J. Similar to our previous work (Ji *et al.*, 2021a), the database owner selects the non-fingerprinted entries and modifies their value by solving a linear programing problem, which is discussed in Section 5.2.

In Section 6, we show that by applying ${\text{Mtg}}_{\text{row}}(S)$ and ${\text{Mtg}}_{\text{col}}(J)$ after our proposed vanilla fingerprinting scheme, the malicious SP can hardly distort large portion of the fingerprint bits anymore, even if it introduces significant utility loss in the database (e.g. by decreasing database accuracy and making the SNP–phenotype associations less consistent compared to the ground-truth measured on the original—non-fingerprinted—database). For example, if the malicious SP compromises around 24% accuracy and 20% consistency in SNP–phenotype associations, it can only distort about 30% fingerprint bits. This implies that the malicious SP will be held responsible for the genomic database leakage with high probability (Ji *et al.*, 2021a).

**Contributions and Broader Impact.** To the best of our knowledge, our work is the first to investigate the liability issue when sharing genomic relational databases (i.e. the collection of genomic data of individuals with the same attributes) and at the same time address the threats of correlation attacks due to the unique biological characteristics.

Our proposed robust genomic database fingerprinting scheme helps facilitate the development of genomic research, which requires large-scale genomic data analyses, and is increasingly relying upon the sharing of genomic databases with various SPs. The ideas developed in this article also shed light on sharing other sensitive biomedical databases, e.g. electrocardiogram and electrooculogram data samples, where the data correlations are determined by the spatio-temporal dependency between data records. In this case, the correlations can be characterized by autocorrelation and cross-correlations or they are modeled as a Markov process. We will also study these types of databases in the future.

**Article** **organization**. We review related works on database fingerprinting schemes in Section 2. In Section 3, we present the system and threat models, and evaluation metrics. Section 4 introduces the foundation of our scheme, i.e. the vanilla fingerprinting scheme for genomic databases. Then, in Section 5, we consolidate this foundation by developing mitigation techniques against various correlation attacks on the genomic database. In Section 6, we show the vulnerabilities of the vanilla fingerprinting scheme for genomic databases, and also demonstrate the performance of mitigation techniques from both database utility and fingerprint robustness perspective. Section 7 discusses the limitations of the proposed scheme and points out future research directions. Finally, Section 8 concludes the article.

## 2 Related work

The seminal work of database fingerprinting is proposed by Agrawal *et al.* (2003), which assumes that the database consumer can tolerate a small amount of error in the marked databases. Then, based on Agrawal *et al.* (2003), some variants have been proposed (Guo *et al.*, 2006; Li *et al.*, 2005; Liu *et al.*, 2004). For instance, Li *et al.* (2005) develop a database fingerprinting scheme by extending Agrawal *et al.* (2003) to enable the insertion and extraction of arbitrary bit-strings in relations. However, these works do not consider the correlations among data entries, which makes them vulnerable to correlation attacks (Ji *et al.*, 2021a). Records in Genomic databases usually have much stronger correlations caused by Mendel’s law and linkage disequilibrium, which make the genomic database prone to correlation attacks.

Recently, some works have explicitly taken the genomic data correlations into account in fingerprinting scheme design. In particular, Yilmaz and Ayday (2020) develop a probabilistic fingerprinting scheme by considering the conditional probabilities between genomic data points of a single individual. Ayday *et al.* (2019) propose an optimization-based fingerprinting scheme for sharing personal genomic sequential data by jointly considering collusion attack and data correlation. However, these two works focus on the genomic data of an individual, instead of a genomic database, where individuals may have kinship relationships. Öksüz *et al.* (2021) develop a watermarking scheme for sequential genomic data based on belief propagation, which considers the privacy of data and the robustness of watermark requirements at the same time. Ji *et al.* (2021a) propose mitigation techniques against general correlation attacks targeted on generic relational databases and show that the proposed technique can be applied after any existing relational database fingerprinting scheme to achieve robustness against correlation attacks.

However, the above- mentioned works cannot be directly applied to genomic database fingerprinting, because they fail to consider the characteristics that are unique to the genomic data. Particularly, (i) hereditary units governed by the Mendel’s law can be utilized by the malicious SP to further infer the potentially fingerprinted locations. (ii) Limited values of genomic data also make the utility-preserving fingerprinting a challenging task. Thus, in this article, we first show the vulnerability of genomic database fingerprinting against correlation attacks that take advantage of the Mendel’s law and linkage disequilibrium. Then, we discuss how to mitigate the identified attacks in a way that the utility of the database is preserved.

## 3 System, threat model and success metrics

Now, we discuss the genomic database fingerprinting system, the considered various threats, and fingerprint robustness and utility metrics.

### 3.1 Genomic database fingerprinting system model

We consider a database owner (Alice) with a genomic database [e.g. dbSNP (Wheeler *et al.*, 2008)] including SNPs of a certain population, i.e. each row corresponds to the SNP sequence of a specific individual. Each individual has two alleles for each SNP position, and each of these alleles is inherited from one of their parents. Thus, each SNP (i.e. each entry of the database) can be represented by the number of its minor alleles as 0, 1 or 2, and can be encoded as ‘00’, ‘01’ or ‘10’, respectively. In this article, we focus on sharing SNP databases, because such databases are critical to many genomic and medical research (Mitchell *et al.*, 2004), e.g. genome-wide association studies (GWASs) (Carlson *et al.*, 2003). The techniques developed in this article can be applied to other types of genomic databases (e.g. ones including nucleotides that may contain four values A, G, C or T) by simply changing the data coding.

**Fig. 1.**
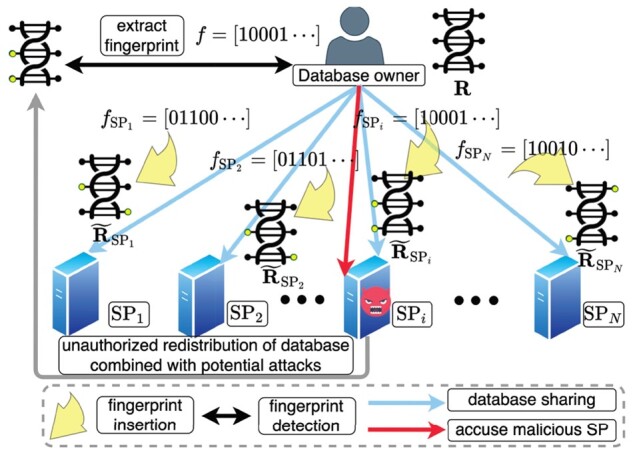
The genomic database fingerprinting system, where Alice adds a unique fingerprint in each copy of her genomic database **R** when sharing. The inserted fingerprint will change entries at different locations (indicated by the yellow dots) in **R**. She is able to identify the malicious SP who pirates and redistributes her database using the customized fingerprint

We present the system model for genomic database fingerprinting in Figure 1. We denote the genomic database owned by Alice as **R**. When Alice wants to share the database with various medical SPs, she includes a unique fingerprint in each copy of her database. The fingerprint bit-string customized for each SP is a randomly generated binary bit-string (elaborated in Section 4.1). The fingerprint essentially changes different entries in **R** at various positions (indicated by the yellow dots). The fingerprint generated for the *i*th SP (${\text{SP}}_{i}$) is $f_{{\text{SP}}_{i}}$, and the fingerprinted genomic database received by ${\text{SP}}_{i}$ is represented as ${\tilde{R}}_{f_{{\text{SP}}_{i}}}$. We also use $\tilde{R}$ to represent a generic fingerprinted genomic database.

In real-world applications, some of the SPs may be malicious (e.g. ${\text{SP}}_{i}$ in Fig. 1) who will redistribute its received genomic database copy after conducting certain attacks (discussed in Section 3.2) on top of it.

**System with** **vanilla** **fingerprinting.** If ${\text{SP}}_{i}$ compromise ${\tilde{R}}_{f_{{\text{SP}}_{i}}}$ via random bit flipping attack, Alice is able to identify ${\text{SP}}_{i}$ as the traitor by extracting a large portion of its fingerprint from the leaked database by only using the proposed vanilla fingerprint scheme (Section 4). However, if ${\text{SP}}_{i}$ launches correlation-based attacks on ${\tilde{R}}_{f_{{\text{SP}}_{i}}}$, it can avoid being accused of data leakage with large probability.

**System with** **robust** **fingerprinting.** The correlation-based attacks can be effectively mitigated, if ${\tilde{R}}_{f_{{\text{SP}}_{i}}}$ is generated by adopting our proposed robust fingerprinting scheme, and thus, ${\text{SP}}_{i}$ will still be held responsible for illegal redistribution. We will empirically evaluate these using a real-world genomic database in Section 6.

### 3.2 Threat model

Fingerprinted databases are subject to various attacks summarized in the following. Note that in all considered attacks, a malicious SP can change/modify most of the entries in $\tilde{R}$ to distort the fingerprint (and to avoid being accused). However, such a pirated database will have significantly poor utility measured in terms of database accuracy and consistency of SNP–phenotype association (see Section 3.4). Thus, a rational SP will try to get away with making pirated copies of the genomic databases by changing as few entries as possible in order to maintain high utility for the pirated database and gain illegal profit.

**Random** **bit** **flipping** **attack.** In this attack, to pirate a database, a malicious SP selects random entries in its received copy of the genomic database and flips their bit values (Agrawal *et al.*, 2003). For example, a SNP value 2 (‘10’) becomes 0 (‘00’) after the attack.

**Row-wise correlation attack**  ${\text{Atk}}_{\text{row}}(S)$. As discussed in Section 1, a malicious SP may utilize Mendel’s law and similarities among family members’ genomes to infer the potentially changed loci in the fingerprinted database. Thus, we assume that the malicious SP has access to the sets of families in the database as well as the genome similarities (denoted as S) among family members in each set. Note that this is a valid assumption, because quite a few works have shown that kinship or familial relationships from SNP genotyping data can be inferred with very high accuracy for small and medium size groups [e.g. dozens or hundreds of individuals (Goudet *et al.*, 2018; Park *et al.*, 2013), large size populations (Wang *et al.*, 2017), or even worldwide (Li *et al.*, 2008)] or such information can be obtained directly from the metadata.

Thus, upon receiving a fingerprinted copy of the genomic database, the malicious SP will check all SNPs at the same loci of family members and then flips SNPs at the loci that violate Mendel’s law. For example, if both parents have SNP value 0, but their children have SNP value 1 at the same locus, then, the malicious SP knows that one or more family members’ SNP values have been changed with high probability due to fingerprint insertion, since such change can be due to a mutation with a slight probability. Next, the malicious SP can further calculates the empirical row-wise correlations (denoted as S′) from the received fingerprinted copy, compares S′ with S, and changes entries that leads to large discrepancy between them.

**Column-wise correlation attack**  ${\text{Atk}}_{\text{col}}(J)$. We model the publicly known allelic associations (linkage disequilibrium) between SNP values at different loci as a set of joint distributions J. Once a malicious SP receives the fingerprinted database, it can calculate a new set of empirical pair-wise joint distributions J′. Then, it compares J′ and J, and flips the entries in the fingerprinted copy that leads to large discrepancy between them.

In this article, we do not consider other common attacks, such as the subset attack and superset attack (Li *et al.*, 2005), because they are usually much weaker than the random bit flipping attack as shown in Yilmaz and Ayday (2020). Another widely investigated attack is the collusion attack (Boneh and Shaw, 1998, 1995; Pfitzmann and Waidner, 1997). Our proposed robust fingerprinting scheme for genomic databases can also be extended to collusion-resistant genomic database fingerprinting by incorporating collusion-resistant codewords when generating the fingerprint (Boneh and Shaw, 1995). We will extend our work in the scenario of colluding medical SPs in future.

### 3.3 Fingerprint robustness metrics

The primary goal of a malicious SP is to distort the fingerprint in its received copy to avoid being accused. We use the percentage of compromised fingerprint bits, i.e. ${\text{Per}}_{\text{cmp}}$, to measure the robustness of the fingerprint scheme. ${\text{Per}}_{\text{cmp}}$ calculates the percentage of mismatches between the fingerprint bit-sting extracted from the compromised fingerprinted database and the original fingerprint bit-string that is used to generate the fingerprinted genomic database. In our previous work (Ji *et al.*, 2021a), we have shown that if the malicious SP can compromise more than 50% of the fingerprint bits, it can cause the database owner to accuse other innocent SPs who also received the databases. In this article, we only focus on ${\text{Per}}_{\text{cmp}}$, because other robustness metrics (e.g. the accusable ranking of a malicious SP) directly depends on ${\text{Per}}_{\text{cmp}}$ (Ji *et al.*, 2021a).

### 3.4 Utility metrics

Fingerprinting naturally changes the content of databases (i.e. the values of the SNPs), and hence degrades its utility. We quantify the utility of a fingerprinted genomic database using the following metrics.

**Accuracy of the database, i.e.** ***Acc*.** It calculates the percentage of matched data entries between the original genomic database and the fingerprinted copy (or the compromised fingerprinted copy, i.e. the pirated copy generated by a malicious SP). The higher *Acc*, the fewer entries are changed due to fingerprint insertion, attack from the malicious SP, or mitigation to resist the attacks, and thus, the higher the utility.

**Consistency of SNP–phenotype association.** GWAS is a widely adopted method to identify genetic variations that are associated with a particular phenotype (e.g. a disease). In GWAS, a researcher usually quantifies the associations between a phenotype and each SNP in the database using a *P*-value with a confidence level of 95% (Halimi *et al.*, 2021; Sheskin, 2003). In particular, SNPs with low *P*-values (typically smaller than 0.05) are considered to have strong associations with the phenotype (i.e. the association cannot be due to chance). In general, a larger utility loss in terms of accuracy degradation will lead to less accurate SNP–phenotype association. To evaluate the *P*-value of each SNP in the genomic database, we first randomly divide the database into non-overlapping case (denoted as *S*) and control (denoted as *C*) groups, and then follow the steps listed in (1) to perform the calculations.

(1) $$\begin{array}{c} OR=\frac{C_{0}(S_{1}+S_{2})}{S_{0}(C_{1}+C_{2})},\\ S\mathrm{tdErr}(\ln(OR))\\ =\sqrt{\frac{1}{S_{1}+S_{2}}+\frac{1}{S_{0}}+\frac{1}{C_{1}+C_{2}}+\frac{1}{C_{0}}},\\ z=\frac{\ln(OR)}{\mathrm{StdErr}(\ln(OR))},\\ p=\Psi (-z)+1-\Psi (z). \end{array}$$

In particular, in (1), OR is the odd ratio, *C*_0_, *C*_1_ and *C*_2_ (or *S*_0_, *S*_1_ and *S*_2_) are the numbers representing a specific SNP taking a value of 0, 1 and 2 in the control (or case) group. StdErr(ln(OR)) is the standard error of the logarithm of the odd ratio, and *z* is interpreted as the standard normal deviation (i.e. *z*-value). Finally, the *P*-value is the area (probability) of the normal distribution that falls outside ±z, and it can be obtained using Ψ(·); the cumulative distribution function of the standard normal distribution.

To evaluate the utility of the genomic database, we identify the top-50 SNPs (i.e. the 50 SNPs with the lowest *P*-values) from the original (non-fingerprinted) database. Then, we check how many of such SNPs are preserved (i.e. remains to be the top-50 SNPs) after fingerprinting or various attacks. Note that we only consider the consistency of SNP–phenotype association for individual SNPs (i.e. not the consistency of SNP–phenotype association for SNP-tuples). This is because the proposed mitigation techniques can preserve the Pearson’s correlations among each pair of SNPs (see Section 5.2 for details).

## 4 The foundation: vanilla genomic fingerprinting scheme

Now, we establish the foundation of our robust fingerprinting scheme for genomic database. Our developed vanilla scheme is inspired by Li *et al.* (2005), which enables the insertion and extraction of arbitrary bit-strings in databases. However, our scheme differs from Li *et al.* (2005) and its variants, e.g. Guo *et al.* (2006) and Ji *et al.* (2021a), as they only mark one bit position in each selected row, which leads to less fingerprinting robustness due to significantly larger number of attributes (e.g. number of SNPs in a genomic database) and strong correlation patterns in genomic data.

### 4.1 Fingerprint insertion phase of the vanilla scheme

When the database owner shares a fingerprinted copy of the genomic database **R** with a SP (whose id is *n*), it first generates the fingerprint bit-string $f_{{\text{SP}}_{n}}=\mathrm{Hash}(K|n)$, where K is the secret key of the database owner and | stands for the concatenation operator [in this article, we use MD5 to generate a 128-bits fingerprint string, because if the database owner shares *C* copies of its database, then as long as L≥lnC, the fingerprinting mechanism can thwart exhaustive search and various types of attacks, and in most cases a 64-bits fingerprint string is shown to provide high robustness (Li *et al.*, 2005)]. The database owner uses a cryptographic pseudorandom sequence generator U to select specific bit positions of specific SNPs from some individuals and fingerprint these bits using mark bits *m’*s, which are the result of the XOR operation between the random mask bits (*x’*s) and randomly selected fingerprint bits (*f_l’_*s), i.e. $m=x\;⊕\;f_{l}$, where *f_l_* is the *l*th bit in $f_{{\text{SP}}_{n}}$.

To be more specific, for all individuals in the genomic database, the database owner fingerprints the SNP sequence if $U_{1}(K|r_{i}.\text{primary}\;\text{key})\;\text{mod}\;\left\lfloor 1/\gamma_{r}\right\rfloor =0$, where $\gamma_{r}\in (0,1)$ is the row fingerprint density. As a result, the fraction of fingerprinted SNP sequences in **R** is approximately *γ_r_*. For all SNPs in a selected sequence (i.e. $r_{i}$), the element with attribute *p* (i.e. the SNP value at loci *p* of $r_{i}$ represented by $r_{i}[p]$) will be fingerprinted if $U_{2}(K|r_{i}.\text{primary}\;\text{key}|p)\;\text{mod}\;\left\lfloor 1/\gamma_{l}\right\rfloor =0$, where $\gamma_{l}\in (0,1)$ is the column fingerprint density. Then, the database owner sets the binary mask bit $x=U_{3}(K|r_{i}.\text{primary}\;\text{key}|p)\;\text{mod}\;2$, and selects one bit position of $f_{{\text{SP}}_{n}}$ via $l=(U_{4}(K|r_{i}.\text{primary}\;\text{key}|p)\;\text{mod}\;L)+1$. Next, it obtains the mark bit *m* as $m=x\;⊕\;f_{{\text{SP}}_{n}}[l]$, and selects a bit position (count backwards) of $r_{i}[p]$ via $t=(U_{5}(K|r_{i}.\text{primary}\;\text{key}|p)\;\text{mod}\;2)+1$. Finally, it fingerprints $r_{i}[p]$ by replacing the *t*th to the last bit of $r_{i}[p]$ with *m*. We summarize the steps of the fingerprint insertion phase of the vanilla fingerprinting scheme in Algorithm 1.

Algorithm 1: Vanilla scheme: fingerprint insertion

 **Input:** The original genomic relational database **R**, row fingerprinting density *γ_r_*, column fingerprinting density *γ_l_*, database owner’s secret key K, pseudorandom number sequence generator U and the SP’s series number *n* (which can be public).

 **Output:** The vanilla fingerprinted genomic relational database **R**.

1 Generate the fingerprint bit-string of SP *n*, i.e. $f_{{\text{SP}}_{n}}=\mathrm{Hash}(K|n)$;

2 **forall** *individual* $r_{i}\in R$  **do**

3 **if**  $U_{1}(K|r_{i}.\text{primary}\;\text{key})\;\text{mod}\;\left\lfloor 1/\gamma_{r}\right\rfloor =0$  **then**

4  //fingerprint the SNP sequence of the *i*th individual

5  **forall** *SNP element* $r_{i}[p]\in r_{i}$  **do**

6   **if**  $U_{2}(K|r_{i}.\text{primary}\;\text{key}|p)\;\text{mod}\;\left\lfloor 1/\gamma_{l}\right\rfloor =0$  **then**

7  //fingerprint the *p*th SNP of the *i*th individual

8   Set mask_bit x=0, if $U_{3}(K|r_{i}.\text{primary}\;\text{key}|p)$ is even; otherwise set *x *=* *1.

9     

$$\text{fingerprint}\_\text{index}\;l=U_{4}(K|r_{i}.\text{primary}\;\text{key}|p)\;\text{mod}\;L.$$

10     

$$\text{fingerprint}\_\text{bit}\;f_{l}=f_{{\text{SP}}_{n}}(l).$$

11     

$$\text{mark}\_\text{bit}\;m=x\;⊕\;f_{l}.$$

12   Set $t=(U_{5}(K|r_{i}.\text{primary}\;\text{key}|p)\;\text{mod}\;2)+1$.

13   Set the *t*th to the last bit of $r_{i}[p]$ to *m*.

Different from Li *et al.* (2005), Guo *et al.* (2006) and Ji *et al.* (2021a), by involving *γ_l_*, our vanilla scheme can mark more bits in each selected row. In our recent work (Ji *et al.*, 2021b), we also derived a closed-form expression to characterize the relationship between fingerprint robustness and database utility. Thus, by jointly tuning *γ_r_* and *γ_l_*, we can also achieve desired tradeoff between robustness and utility. We will theoretically and empirically investigate this in the future.

### 4.2 Fingerprint extraction phase of the vanilla scheme

When the database owner observes a leaked (or pirated) genomic database denoted as $\bar{R}$, it tries to identify the traitor (i.e. the malicious SP) by extracting the fingerprint from $\bar{R}$ and comparing it with the fingerprints of all SPs who have received its genomic database.

We present the fingerprint extraction phase of the vanilla scheme in Algorithm 2. Specifically, the database owner first initiates a fingerprint template $f=(f_{1},f_{2},\ldots ,f_{L})=(?,?,\ldots ,?)$. Here, ‘?’ means that the fingerprint bit at that position remains to be determined [similar symbol has also been used in other works (Agrawal *et al.*, 2003; Boneh and Shaw, 1995; Ji *et al.*, 2021a; Li *et al.*, 2005)]. Then, the database owner determines the mask bit (*x*), obtains the corresponding indices of fingerprint bits (*l’*s), locates the bit positions of the fingerprinted SNP elements exactly as in the fingerprint insertion phase, and finally fills in each ‘?’ using a voting scheme. To be more precise, for each fingerprinted SNP $\bar{r_{i}}[p]$, the database owner obtains the corresponding mark bit *m* by reading the *t*th to the last bit of $\bar{r_{i}}[p]$, and recovers one instance of the *l*th bit of the fingerprint bit-string via $f_{l}=m\;⊕\;x$. Since the value of *f_l_* may be changed by the malicious SP, the database owner maintains and updates two counting arrays $c_{0}$ and $c_{1}$, where $c_{0}(l)$ and $c_{1}(l)$ record the number of times *f_l_* is recovered as 0 and 1, respectively. Finally, the database owner sets f(l)=1 if $c_{1}(l)/(c_{1}(l)+c_{0}(l))\geq \tau$, and f(l)=0 if $c_{0}(l)/(c_{1}(l)+c_{0}(l))\geq \tau$, otherwise f(l)=? (i.e. this fingerprint bit cannot be determined due to the database owner’s low confidence), where τ∈(0.5,1] is the parameter that quantifies the database owner’s confidence in the fingerprint recovery phase [in this article, we set τ=0.7, which implies the database owner has higher confidence during fingerprint extraction than the other works, e.g. in Ji *et al.* (2021a) and Li *et al.* (2005), *τ* is slightly higher than 0.5].

Algorithm 2: Vanilla scheme: fingerprint extraction

**Input:** The leaked genomic database $\bar{R}$, row fingerprinting density *γ_r_*, column fingerprinting density *γ_l_*, database owner’s secret key K, pseudorandom number sequence generator U and a fingerprint template (?,?,…,?), where ? represents unknown value.

**Output:** The extracted fingerprint bit-string **f** from the leaked database.

1 Initialize $c_{0}(l)=c_{1}(l)=0,\forall l\in [1,L]$.

**2 forall** *individual* $\bar{r_{i}}\in \bar{R}$  **do**

**3  if**  $U_{1}(K|\bar{r_{i}}.\text{primary}\;\text{key})\;\text{mod}\;\left\lfloor 1/\gamma_{r}\right\rfloor =0$  **then**

**4   forall** *SNP element* $\bar{r_{i}}[p]\in r_{i}$  **do**

**5    if**  $U_{2}(K|\bar{r_{i}}.\text{primary}\;\text{key}|p)\;\text{mod}\;\left\lfloor 1/\gamma_{l}\right\rfloor =0$  **then**

6    Set mask_bit x=0, if $U_{3}(K|\bar{r_{i}}.\text{primary}\;\text{key}|p)$ is even; otherwise set *x *=* *1.

7    $\text{fingerprint}\_\text{index}\;l=U_{4}(K|\bar{r_{i}}.\text{primary}\;\text{key}|p)\;\text{mod}\;L.$

8    Set $t=(U_{5}(K|\bar{r_{i}}.\text{primary}\;\text{key}|p)\;\text{mod}\;2)+1$.

9    Set the mark_bit  *m* as the *t*th to the last bit of $\bar{r_{i}}[p]$.

10   Recover the fingerprint bit $f_{l}=m\;⊕\;x$.

11   $c_{1}(l)++$, if *f_l_* = 1; otherwise $c_{0}(l)++$.

**12 forall**  l∈[1,L]  **do**

13 f(l)=1, if $c_{1}(l)/(c_{1}(l)+c_{0}(l))\geq \tau$, and f(l)=0, if $c_{0}(l)/(c_{1}(l)+c_{0}(l))\geq \tau .$

## 5 Consolidating the foundation: making the vanilla genomic fingerprinting scheme robust against correlation attacks

Here, we propose a robust fingerprinting scheme for genomic databases against the correlation attacks identified in Section 3.2. The robust scheme is developed by augmenting the vanilla scheme using two mitigation techniques, which can serve as the post-processing steps after the vanilla fingerprinting. It brings us two benefits: (i) fingerprint robustness of the vanilla scheme is maintained, because the devised mitigation techniques only change non-fingerprinted entries and (ii) the mitigation techniques can be applied to any vanilla fingerprinting schemes to resist correlation attacks on genomic databases. As discussed in Section 4.1, we choose our developed vanilla scheme to have more control over the fingerprint density on each selected SNP sequence. In practice, one can develop their own vanilla scheme depending on the content of their genomic database and then apply our proposed mitigation techniques to make their scheme also robust against the correlation attack.

In high level, to provide robustness against the row-wise correlation ${\text{Atk}}_{\text{row}}(S)$ and column-wise correlation attack ${\text{Atk}}_{\text{col}}(J)$, the database owner (Alice) will perform mitigation steps (after the vanilla fingerprinting) by utilizing Mendel’s law and her prior knowledge S (correlation of genomic data among different individuals), J (correlation of SNP values at different loci) to reduce the discrepancy caused by fingerprinting insertion. We will show that to implement the proposed mitigation steps, Alice needs to change only a few entries after the vanilla fingerprinting (e.g. <3% as shown in Table 3 in Section 6.3).

**Table 3.** Additional change caused by mitigation

*γ_r_γ_l_*	0.05 (%)	0.06 (%)	0.07 (%)	0.08 (%)	0.09 (%)	0.1 (%)
0.05	2.87	2.89	2.91	3.10	3.02	3.05
0.06	2.89	2.82	2.85	2.98	2.97	2.99
0.07	2.86	2.85	2.92	3.06	3.00	3.03
0.08	2.93	2.95	2.98	3.17	3.09	3.20
0.09	2.87	2.87	2.91	3.13	2.89	3.10
0.1	2.98	2.95	3.07	3.29	3.17	3.36

### 5.1 Mitigating the row-wise correlation attack

To mitigate the row-wise correlation attack (in Section 3.2), we develop ${\text{Mtg}}_{\text{row}}(S)$, which is composed of two phases. The first phase tries to eliminate all SNP loci that violate the Mendel’s law, and the second phase makes the similarities of genome data among family members close to that before the fingerprint (generated by the vanilla scheme) is inserted. In particular, for each family set denoted as $\text{fmly},\;{\text{Mtg}}_{\text{row}}(S)$ checks all fingerprinted SNPs of all family members. If the SNP-tuple at a locus violates the Mendel’s law, ${\text{Mtg}}_{\text{row}}(S)$ changes other non-fingerprinted entries in the tuple to make them comply with the Mendel’s law. For example, if a mother–father–child SNP-tuple at a specific locus takes value ‘2-1-0’ (2 for mother, 1 for father and 0 for child, which violates the Mendel’s law), and ‘1’ (value of the SNP for the mother) is the fingerprinted SNP, then, ${\text{Mtg}}_{\text{row}}(S)$ can modify this tuple as ‘2-1-1’.

In the second phase, for each family set (i.e. fmly) in the genomic database, Alice changes the SNP values of the family members such that the cumulative similarities between individuals with kinship relation are close to their original similarities. This can be formulated as the following optimization problem:

(2) $$\begin{array}{c} \min_{\tilde{\tilde{r_{i}}}}\;\sum_{i,j\in \text{fmly}}\;|s_{ij}{}^{\text{fmly}}-{\tilde{\tilde{s_{ij}}}}^{\text{fmly}}|\\ s.t.\;\tilde{\tilde{r_{i}}}=\text{value}\;\text{change}(\tilde{r_{i}}),\\ {\tilde{\tilde{s_{ij}}}}^{\text{fmly}}=\langle \tilde{\tilde{r_{i}}},\tilde{\tilde{r_{j}}}\rangle . \end{array}$$

In (2), $s_{ij}{}^{\text{fmly}}$ stands for the publicly known similarities between two family members in fmly and ${\tilde{\tilde{s_{ij}}}}^{\text{fmly}}$ stands for the similarities after row-wise mitigation [in this article, the similarity between two individuals is defined as the inner product between their SNP sequences. One can also define the similarity using a common metric adopted in biology, e.g. the allele shared distance (i.e. ASD) (Park *et al.*, 2013), which is also related to the considered inner product]. We denote the SNP sequence of an individual *i* after the mitigation as $\tilde{\tilde{r_{i}}}$. Also, value change(·) is the function that changes each SNP attribute of $\tilde{r_{i}}$, and it will be elaborated later. (2) is an integer programing problem. Since each SNP sequence may contain thousands of SNPs, it will be computationally expensive to obtain the optimal value of $\tilde{\tilde{r_{i}}}$. Thus, we solve it heuristically. Particularly, if $s_{ij}{}^{\text{fmly}}>{\tilde{s_{ij}}}^{\text{fmly}}$ (${\tilde{s_{ij}}}^{\text{fmly}}$ is the empirical similarity calculated after the vanilla fingerprinting), the database owner needs to post-process $\tilde{r_{i}},\forall i\in \text{fmly}$ to increase the similarity. On the other hand, if $s_{ij}{}^{\text{fmly}}<{\tilde{s_{ij}}}^{\text{fmly}}$, the database owner needs to post-process $\tilde{r_{i}},\forall i\in \text{fmly}$ to decrease the similarity.

We use the example of SNP sequences from mother–father–child to further explain how to increase or decrease the similarity. To increase ${\tilde{s_{ij}}}^{\text{fmly}}$, we randomly select a certain number of non-fingerprinted 3-SNP-tuples (i.e. mother–father–child) with value ‘0-0-0’ and then change it to ‘1-0-1’ or ‘0-1-1’ depending on whether ${\tilde{s_{ij}}}^{\text{fmly}}$ is a mother–child or father–child similarity. We only change 3-SNP-tuples with value ‘0-0-0’, because this is the one of the most common 3-tuples in all families, and modification of mother–child tuples will not have an impact on the father–child similarity (vice-versa). To decrease ${\tilde{s_{ij}}}^{\text{fmly}}$, we let the database owner change a certain number of 3-tuples with value ‘1-0-1’ (or ‘0-1-1’) to ‘0-0-0’ if ${\tilde{s_{ij}}}^{\text{fmly}}$ is the mother–child (or father–child) similarity. The reasons are exactly the same as the case of increasing ${\tilde{s_{ij}}}^{\text{fmly}}$. Although there are also higher degrees of relatedness among family members (e.g. the SNP correlations between grandparents and grandchildren), those correlation (or similarity) are usually much weaker than the first order correlations (e.g. mother–father–child). The vanilla fingerprinting is subject to destroying the strong correlations the most (and hence an attacker can easily infer the fingerprint due to the distorted correlations). For higher degree family members, the original correlations are not high and fingerprint will not destroy such correlation too much. We will experimentally verify this in Section 6.1.

Note that the row-wise mitigation technique for genomic databases is different than the one developed for general databases in our previous work (Ji *et al.*, 2021a), which changes entries of non-fingerprinted data records to make the newly obtained similarities as far away from Alice’s prior knowledge S to mislead the malicious SP. In contrast, here, we make the new similarities close to S in order to alleviate ${\text{Atk}}_{\text{row}}(S)$ (try to make ${\text{Atk}}_{\text{row}}(S)$ distort less fingerprint bits), and if the objective function on (2) equals 0, ${\text{Atk}}_{\text{row}}(S)$ is completely invalidated. The reason that we can pursue this in genomic database is because each row has much more attributes then general databases and the number of unique values is small (i.e. only three options: 0, 1 and 2). Besides, the row-wise mitigation techniques developed in Ji *et al.* (2021a) solves an NP-hard combinatorial search problem greedily, which introduces large computation overhead.

### 5.2 Mitigating column-wise correlation attack

To make the vanilla scheme robust against column-wise correlation attack, we propose ${\text{Mtg}}_{\text{col}}(J)$, which transforms the vanilla fingerprinted genomic database to have column-wise joint distributions (e.g. linkage disequilibrium between the SNPs) close to the publicly known joint distributions in J. Inspired by Ji *et al.* (2021a), we develop ${\text{Mtg}}_{\text{col}}(J)$ using the idea of ‘optimal transport’ (Cuturi, 2013), which moves the probability mass of the marginal distribution of each SNP attribute of the vanilla fingerprinted genomic database to resemble the distribution obtained from the marginalization of each reference joint distribution in J. Then, the optimal transport plan is used to change the entries in the genomic database after the vanilla fingerprinting.

In particular, for a specific SNP (column, i.e. locus of SNP sequence) *p*, we denote its marginal distribution obtained after the vanilla fingerprinting as $\mathrm{Pr}(C_{\tilde{p}})$, and that obtained from the marginalization of a joint distribution $J_{p,q}$ distribution in J as $\mathrm{Pr}(C_{p})=J_{p,q}1^{T}$ (here *q* can be any attribute that is different from *p*, because the marginalization with respect to *p* using different $J_{p,q}$ will lead to the identical marginal distribution of *p*). To move the mass of $\mathrm{Pr}(C_{\tilde{p}})$ to resemble $\mathrm{Pr}(C_{p})$, we need to find another joint distribution (i.e. the mass transport plan) $G_{\tilde{p},p}\in R^{3\times 3}$, whose marginal distributions are identical to $\mathrm{Pr}(C_{\tilde{p}})$ and $\mathrm{Pr}(C_{p})$. Then, $G_{\tilde{p},p}(a,b),a,b\in \{0,1,2\}$ indicates that the database owner should change $G_{\tilde{p},p}(a,b)$ percentage of entries in the vanilla fingerprinted genomic database whose attribute *p* (SNP *p*) takes value *a* to value *b*, so as to make $\mathrm{Pr}(C_{\tilde{p}})$ close to $\mathrm{Pr}(C_{p})$. In practice, such a transport plan can be obtained by solving a regularized optimal transport problem, i.e. the entropy regularized Sinkhorn distance minimization (Cuturi, 2013) as follows:

(3) $$d(\mathrm{Pr}(C_{\tilde{p}}),\mathrm{Pr}(C_{p}),\lambda_{p})=\min_{G_{\tilde{p},p}}{\langle G_{\tilde{p},p},\Theta_{\tilde{p},p}\rangle }_{F}-\frac{H(G_{\tilde{p},p})}{\lambda_{p}},$$

where $G_{\tilde{p},p}\in G(\mathrm{Pr}(C_{\tilde{p}}),\mathrm{Pr}(C_{p}))=\{G\in R^{k_{p}\times k_{p}}|G1=\mathrm{Pr}(C_{\tilde{p}}),G^{T}1=\mathrm{Pr}(C_{p})\}$ is the set of all joint probability distributions whose marginal distributions are the probability mass functions of $\mathrm{Pr}(C_{\tilde{p}})$ and $\mathrm{Pr}(C_{p})$. ${\langle \cdot ,\cdot \rangle }_{F}$ denotes the Frobenius inner product of two matrices. Also, $\Theta_{\tilde{p},p}$ is the transport cost matrix and $\Theta_{\tilde{p},p}(a,b)>0$ representing the cost to move a unit percentage of mass from $\mathrm{Pr}(C_{\tilde{p}}=a)$ to $\mathrm{Pr}(C_{\tilde{p}}=b)$. Finally, $H(G_{\tilde{p},p})=-{\langle G_{\tilde{p},p},\;\text{log}\;G_{\tilde{p},p}\rangle }_{F}$ calculates the information entropy of $G_{\tilde{p},p}$ and $\lambda_{p}>0$ is a tuning parameter. In practice, (4) can be efficiently solved by linear programing (Cuturi, 2013). The obtained $G_{\tilde{p},p}$ is more heterogeneous for larger values of *λ_p_*, i.e. the database owner will change less entries after the vanilla fingerprinting, which preserves more database utility. On the contrary, $G_{\tilde{p},p}$ is more homogeneous for smaller values of *λ_p_*, i.e. it causes more SNP entries to be changed, which leads to more utility loss. Although (4) processes each column (attribute) of the genomic database independently, as shown in Ji *et al.* (2021a), the post-processed fingerprinted database will have the Pearson’s correlations among attribute pairs that are close to the prior knowledge J. This further suggests that the mitigation step can boost the utility of the fingerprinted genomic databases.

**Mitigation against additional auxiliary information.** The developed row- and column-wise mitigation techniques focus on the correlation attacks that use generic correlations among genome data. In some task-dependent applications, the malicious SP can also use specific auxiliary information, e.g. race-specific information determined by genome and population structure, to compromise the fingerprinted database. This can be alleviated by involving additional mitigation steps before ${\text{Mtg}}_{\text{row}}(S)$ and ${\text{Mtg}}_{\text{col}}(J)$ to make the vanilla fingerprinted database also match those auxiliary information. More discussion on the availability of these information to the database owner is deferred to Section 7.

## 6 Experiment results

In this section, we first show that the vanilla fingerprinting scheme can resist random bit flipping attacks, but it is vulnerable to the correlation attacks developed specific for genomic databases. The correlation attacks are more powerful, as they can easily distort more than half of the embedded fingerprint bits at only a small cost of database utility (i.e. introducing less error and preserving the SNP–phenotype association). Then, we demonstrate that the proposed mitigation techniques can thwart correlation attacks and make the vanilla scheme robust against them. Since the mitigation techniques only change limited entries on top of the vanilla scheme, they also maintain a high utility for the genomic database. More importantly, we show that if the attacker conducts correlation attacks after the proposed robust fingerprinting scheme, it cannot succeed even if at a significant cost of database utility loss. Similar to Ji *et al.* (2021a), since the row-wise correlation attack and mitigation are computationally light and modify less database entries, we let the malicious SP launches ${\text{Atk}}_{\text{row}}(S)$ followed by ${\text{Atk}}_{\text{col}}(J)$ when compromising a fingerprinted database, and let the database owner perform ${\text{Mtg}}_{\text{row}}(S)$ followed by ${\text{Mtg}}_{\text{col}}(J)$ when making a vanilla fingerprinted database robust.

### 6.1 Genomic database description

We use the SNP data belonging to 1500 individuals from the HapMap dataset (Gibbs *et al.*, 2003). Each individual has 156 data points (i.e. SNPs). In this population, there are 150 families, each of which is composed of 3 individuals, i.e. mother, father and child. We assume that both the database owner and the malicious SP know the members of each family and the pair-wise correlations (e.g. linkage disequilibrium) among SNPs (Section 3.2 discusses why this assumption is valid in practice).

**Fig. 2.**
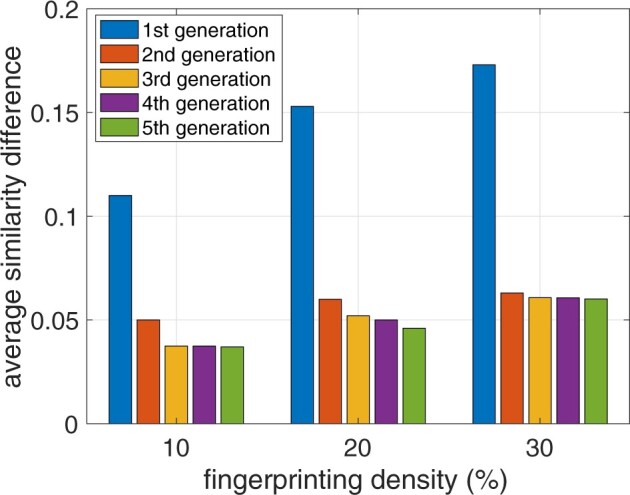
Average absolute value of the SNP cosine similarity difference, before and after fingerprint insertion, among family members and their different generations of simulated descendants

**The importance to consider the correlations due to the first-degree relationships among family members.** As discussed in Section 5.1, row-wise correlations are the strongest among the first-degree family members and the vanilla fingerprinting potentially destroys such strong correlations the most (compared to the correlations between more distant family members). Here, we verify this claim by generating new generations of family members from the offspring of the 150 families and checking the SNP similarities between these new generations and the original 150 pairs of parents before and after fingerprint insertion. In Figure 2, by varying the overall fingerprinting density ($\gamma_{r}\gamma_{l}\in \{10\%,20\%,30\%\}$), we plot the average absolute difference of cosine similarity between the 150 parents and various generations of their descendants due to the vanilla fingerprinting. Clearly, the average change in the similarity with the first generation is the most significant for all considered fingerprinting density, which suggests that mother–father–child trio has the strongest correlation and it provides the most prior knowledge for the malicious SP to launch the row-wise correlation attack, and hence the row-wise mitigation techniques should preserve the correlations between first degree family members as much as possible. Thus, in the following experiment, we focus on the similarity change between individuals and their first generation of descendants during row-wise correlation attack and mitigation.

### 6.2 Vulnerability of vanilla fingerprinting scheme against correlation attack

#### 6.2.1 Performance against correlation attack

**Fig. 3.**
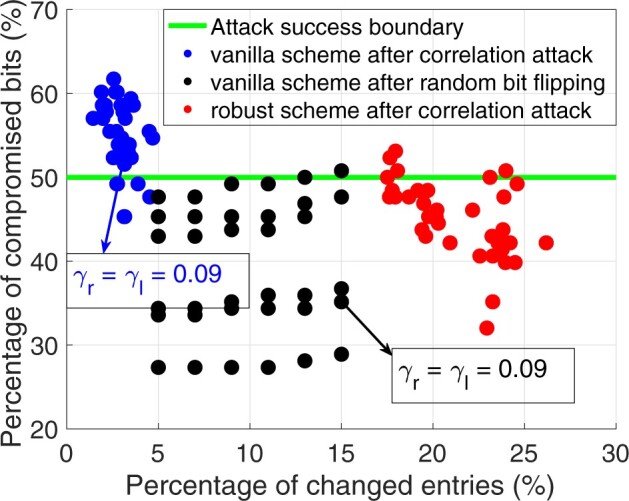
Fingerprint robustness (i.e. percentage of compromised bits) versus utility loss (i.e. percentage of changed entries) when the vanilla scheme is compromised by the correlation attack (blue dots), the vanilla scheme is compromised by the random bit flipping attack (black dots), and the robust scheme is compromised by the correlation attack (red dots). Each dot represents a different experiment. Dots above the attack success boundary (green line) represent a successful attack (in which the database owner incorrectly blames an honest SP for the unauthorized sharing)

We first show the vulnerability of the vanilla scheme under the correlation attacks. To change sufficient number of database entries during fingerprint insertion, we let both row- and column-wise fingerprint density (i.e. *γ_r_* and *γ_l_*) vary in {0.05, 0.06, 0.07, 0.08, 0.09, 0.1}, which gives 36 different fingerprinted databases. Then, we let these databases be compromised by ${\text{Atk}}_{\text{row}}(S)$ followed by ${\text{Atk}}_{\text{col}}(J)$. For each compromised database, we record its percentage of changed entries (i.e. ${\text{Per}}_{\text{chg}}=1-\mathrm{Acc}$) caused by the correlation attack as well as the resulting fingerprint robustness measured in terms of ${\text{Per}}_{\text{cmp}}$ (percentage of compromised fingerprint bits), and scatter the results as blue dots in Figure 3 (we will discuss the black and red dots in the figure in later experiments). As discussed in Section 3.3 and empirically shown in Ji *et al.* (2021a), as long as the malicious SP can compromise more than 50% fingerprint bits, it is able to avoid being detected as the traitor and cause the database owner to accuse other innocent SPs who have also received the database. Thus, we say an attack is successful if ${\text{Per}}_{\text{cmp}}>50\%$, and the green horizontal line in Figure 3 represents the attack success boundary. From Figure 3, we observe that in most of the cases, the identified correlation attacks can compromise more than 50% fingerprint bits (blue points that are above the green line) at the cost of changing only less 5% SNPs in the vanilla fingerprinted database (i.e. an attacker can distort the majority of the fingerprint bits by also keeping the utility of the database high).

In Table 2, we show the consistency of SNP–phenotype association study (discussed in Section 3.4) after the vanilla fingerprinting and the correlation attacks. In particular, we first obtain the set of top-50 SNPs having strong associations with a phenotype (i.e. the 50 SNPs with the lowest *P*-values) from the original (non-fingerprinted) database and denote this set as the ground-truth set. Next, we get the new sets of top-50 SNPs from the vanilla fingerprinted database and the correlation attack-compromised database. Then, we evaluate the consistency by counting the percentage of overlapping SNPs between them and the ground-truth set. From the upper panel of Table 2, we observe that the vanilla fingerprinting preserves high utility of the consistency and the correlation attacks on vanilla fingerprinting scheme also maintain such utility. For example, one of the successful attacks happens when $\gamma_{r}=\gamma_{l}=0.09$ (blue dot indicated by the blue arrow in Fig. 3), and the resultant pirated database copy still preserves more than 96% SNP–phenotype association.

**Table 2.** Consistency of SNP–phenotype association compared with the ground-truth

*P*-value consistency$\gamma_{r}=\gamma_{l}$	0.05 (%)	0.06 (%)	0.07 (%)	0.08 (%)	0.09 (%)	0.1 (%)
Vanilla scheme	100	98	98	98	98	96
Vanilla after corr. attacks	98	96	96	96	96	92
Robust scheme	92	94	94	88	94	92
Robust after corr. attacks	84	92	84	80	86	82

#### 6.2.2 Performance against random bit flipping attack

Next, we compare the attack ability of the random bit flipping attack with our identified correlation attacks. To show the effectiveness of the correlation attacks, we let the random bit flipping attack change more percentage of entries in the vanilla fingerprinted genomic database than the correlation attacks. We also record the fingerprint robustness after the vanilla fingerprinted database is subject to random bit flipping attack. In particular, we set $\gamma_{r}=\gamma_{l}\in \{0.05,0.06,0.07,0.08,0.09,0.1\}$, and let the malicious SP randomly change a certain percentage (i.e. ${\text{Per}}_{\text{chg}}\in \{5\%,7\%,9\%,11\%,13\%15\%\}$) of entries in its received copy of the vanilla fingerprinted genomic database. Thus, we also obtain 36 instances of vanilla fingerprinted databases compromised by random bit flipping attacks. We scatter the recorded results as black dots in Figure 3. Clearly, the random bit flipping attack can hardly compromise more than half of the fingerprint bits if the database owner inserts dense fingerprint in the genomic database, i.e. when $\gamma_{r}=\gamma_{l}\geq 0.06$. In particular, when $\gamma_{r}=\gamma_{l}=0.09$, the malicious SP can only distort 35.16% fingerprint bits at the cost of changing 15% of the SNPs if it launches the random bit flipping attack (indicated by the black arrow in Fig. 3). In contrast, it can compromise 52.34% of the fingerprint bits at the cost of only changing the values of 3.11% of the SNPs if it launches the correlation attacks (indicated by the blue arrow in Fig. 3). This clearly suggests that our vanilla fingerprint scheme developed specially for genomic databases is robust against random bit flipping attacks, but is vulnerable to the attacks using correlations among genome data. Compared with our previous work (Ji *et al.*, 2021a), the identified correlation attacks for genomic data are even more powerful than the ones identified for a general relational database. The reason is that the correlation patterns in genomic data (e.g. Mendel’s law and linkage disequilibrium) are much stronger than the patterns in a general relational database [i.e. census database in Ji *et al.* (2021a)]. Thus, the robust fingerprinting for genomic databases is critical.

### 6.3 Robust genomic database fingerprinting against correlation attacks

Now, we investigate the impact of the proposed robust genomic database fingerprinting scheme. Recall that the robust fingerprinting is achieved by post-process the vanilla fingerprinted genomic database using two mitigation techniques, i.e. ${\text{Mtg}}_{\text{row}}(S)$ and ${\text{Mtg}}_{\text{col}}(J)$.

#### 6.3.1 Impact on database utility

In Table 3, we record the additional percentage of entries being changed due to the post-processing steps (${\text{Mtg}}_{\text{row}}(S)$ and ${\text{Mtg}}_{\text{col}}(J)$). Clearly, as shown in Table 3, the mitigation techniques only need to change about 3% of the SNPs in order to make the post-processed database has row- and column-wise correlation close to S and J and at the same time comply with the Mendel’s law. Thus, the robust fingerprint scheme can preserve high utility of the genomic database, i.e. the database accuracy and the consistency of SNP–phenotype association. For example, as shown in the lower panel of Table 2, in most of the cases, robust scheme achieves more than 90% top-50 SNPs match with the original database.

#### 6.3.2 Impact on fingerprinting robustness

In Figure 3, using red dots, we scatter the fingerprint robustness and the percentage of changed entries when the robust scheme is under the identified correlation attacks (which are identical with that considered in Section 6.2). In particular, comparing with the blue dots, we see that the number of successful attacks (red dots above the green line) is significantly reduced by the proposed mitigation techniques. This suggests that it is very difficult for the malicious SP to compromise more than half of the fingerprint bits using the correlation attacks under the proposed robust scheme, even if the malicious SP has changed more than 20% of the SNPs in the received database. Moreover, correlation attacks on top of the robust fingerprinted genomic database also significantly reduce the utility of SNP–phenotype association study. As shown in Table 2 (the row corresponding to robust after correlation attacks), the percentage of matched top-50 SNPs drops more than 10% compared to the original database. This suggests that the proposed robust genomic database fingerprinting scheme can effectively thwart the identified correlation attacks by just changing about 3% of the SNPs in the post-processing steps and at the same time maintain high database accuracy and consistency of SNP–phenotype association.

### 6.4 Scalability

**Fig. 4.**
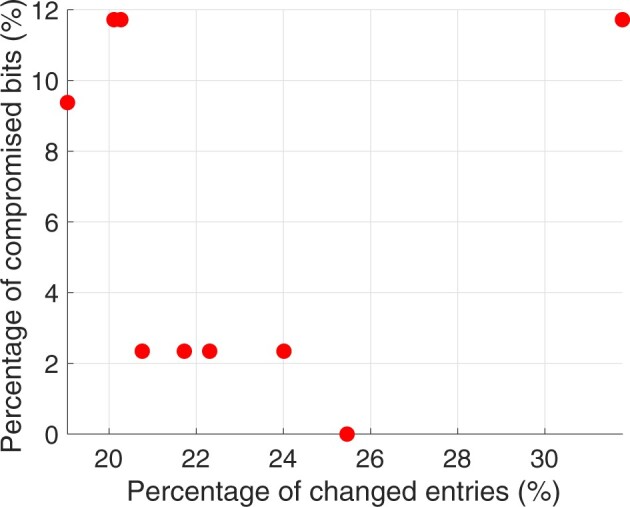
Fingerprint robustness versus utility loss when the proposed robust scheme is compromised by the correlation attacks

**Fig. 5.**
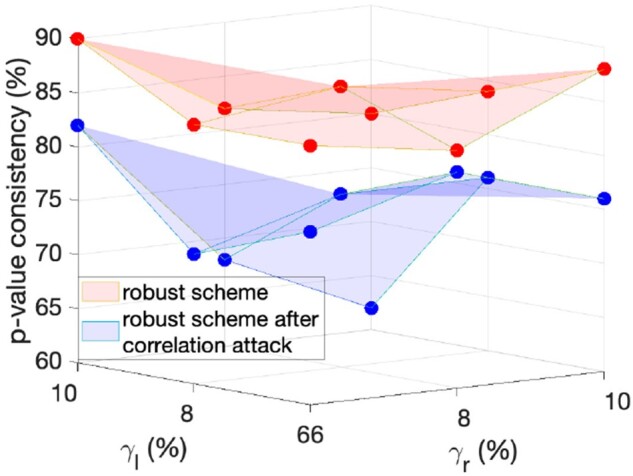
*P*-value consistency before and after the proposed robust scheme is compromised by the correlation attacks

Now, we investigate the performance of the proposed robust fingerprinting scheme for larger genomic databases, where each individual has a higher number of SNPs (i.e. 234). In particular, we consider 8000 individuals among which there are 1333 families (due to the same reasoning in Section 6.1, we also focus on the correlation between mother–child–father tuple). In this experiment, we let $\gamma_{r}=\gamma_{l}\in \{0.06,0.08,0.1\}$. We scatter the pair of percentage of changed entries and percentage of compromised fingerprint bits in Figure 4. We also plot the *P*-value consistency before and after the robust scheme is subject to the identified correlation attacks in Figure 5. From Figure 4, we see that the robustness increases as the database increases (in terms of both rows and columns), because the correlation attacks cannot distort more than 12% of the fingerprint bits even though more than 20% entries are modified. Figure 5 further suggests that if the malicious SP launches the correlation attacks on a robust fingerprinted genomic database, the *P*-value consistency will drop by 10% on average. This experiment shows that our proposed robust fingerprinting scheme is also promising when sharing large genomic databases.

## 7 Discussion

**Independent treatment of elements in SNP sequence.** Note that by checking the change of inner product before and after fingerprinting, (2) essentially treats each element in the SNP sequence independently. However, in practice, blocks of SNP elements may also contain inherent structure, e.g. two or more individuals are identical by descent (IBD) if they have inherited blocks of SNPs from a common ancestor without genetic recombination. Thus, a malicious SP may also use this structural information during an attack. In future work, we will extend the proposed robust fingerprinting scheme to incorporate the recombination of IBD segments during meiosis.

**Limited side-effect of row-wise mitigation.** In the row-wise mitigation, we post-process each pair of first-degree family members in a family set. This may impact the similarity of other pairs in which either individual is involved. For example, updating mother–child pair may increase the similarity of grandfather–grandchild pair. However, as discussed in Section 6.1, such impact is very limited for higher degree family members.

**Assumption on prior knowledge.** To the advantage of the malicious SP, we assume that it has at least equally accurate knowledge about the genomic database (i.e. Mendel’s law, row- and column-wise correlation) compared with the database owner. We do not consider specific auxiliary information (such as SNP population frequencies, rare disease-associated variants, population stratification and SNP–phenotype associations) in this article. If the malicious SP has more auxiliary information than the database owner (which rarely happens in real-world applications), the robustness of the proposed scheme may be compromised. Such robustness degradation will be limited for generic relational database, i.e. the malicious SP still cannot distort more than half of the fingerprint bits (Ji *et al.*, 2021a). We will empirically investigate this for genomic databases by considering various case studies in the future work.

**Privacy concerns in genomic database sharing.** The primary goal of database fingerprinting is to claim copyright and prevent unauthorized redistribution; however, privacy concerns and regulations may also impede genomic data sharing. In our recent work (Ji *et al.*, 2021b), we developed a novel scheme which can leverage the intrinsic randomness introduced by fingerprinting to provide provable privacy guarantees in relational database sharing, i.e. copyright and privacy protection can be achieved in one shot. In future, we will also study privacy-preserving genomic database fingerprinting by adapting the scheme in Ji *et al.* (2021b).

**Computational complexity.** If the genomic database contains *M* individuals, each of which has *N* SNPs, then the computation complexity for ${\text{Mtg}}_{\text{row}}(S)$ is O(MN), because solving (2) requires checking all SNPs of each mother–child–father tuple. The computation complexity for ${\text{Mtg}}_{\text{col}}(J)$ is $O(\frac{3N}{\alpha })$, where 3 is the number of possible instances of SNP values and *α* is the desired error in Sinkhorn-based optimal transport (Le *et al.*, 2021).

## 8 Conclusion

In this article, we have proposed robust fingerprinting for genomic databases composed of SNP sequences. To this end, we first identified the row- and column-wise correlation attack, which utilize Mendel’s law and linkage disequilibrium to distort the embedded fingerprint bits. Next, we developed a vanilla fingerprinting scheme specifically for genomic database by allowing the database owner to embed more fingerprint in each selected SNP sequence. Then, we further made this vanilla scheme robust against the identified correlation attacks by augmenting it with two mitigation techniques, which serve as post-processing steps for the vanilla scheme. In particular, the row-wise mitigation is achieved via solving a cumulative absolute distance minimization, and the column-wise mitigation is realized using optimal mass transport of distributions. Via experiments, we have shown that the identified correlation attacks are much more powerful than common attacks against fingerprinting schemes; they can easily distort more than half of the fingerprint bits at a small cost of database utility. However, these attacks are effectively alleviated by our developed mitigation techniques. The proposed scheme has the potential to further motivate researchers to share their genomic databases with each other, knowing that the shared database is of high utility and the recipient will be hesitant to leak the database due to the provided liability guarantees via the proposed robust fingerprinting scheme.

## Funding

This work was partly supported by the National Library of Medicine of the National Institutes of Health [Award No. R01LM013429]; and the National Science Foundation (NSF) [Grant No. 2050410].

*Conflict of Interest*: none declared.

## References

[btac243-B1] Agrawal R. et al (2003) Watermarking relational data: framework, algorithms and analysis. VLDB J., 12, 157–169.

[btac243-B2] Ayday E. et al (2019) Robust optimization-based watermarking scheme for sequential data. In: *22nd International Symposium on Research in Attacks, Intrusions and Defenses ({RAID} 2019), Beijing China*. pp. 323–336.

[btac243-B3] Boneh D. , Shaw J. (1995) Collusion-secure fingerprinting for digital data. In: *Annual International Cryptology Conference, Santa Barbara, California.* pp. 452–465.

[btac243-B4] Boneh D. , Shaw J. (1998) Collusion-secure fingerprinting for digital data. IEEE Trans. Inform. Theory, 44, 1897–1905.

[btac243-B5] Carlson C.S. et al (2003) Additional SNPs and linkage-disequilibrium analyses are necessary for whole-genome association studies in humans. Nat. Genet., 33, 518–521. 12652300 10.1038/ng1128

[btac243-B6] Cox I.J. et al (1997) Secure spread spectrum watermarking for multimedia. IEEE Trans. Image Process., 6, 1673–1687. 18285237 10.1109/83.650120

[btac243-B7] Cox I.J. et al (2002) Digital Watermarking. Vol. 53. Springer, San Francisco.

[btac243-B8] Cuturi M. (2013) Sinkhorn distances: lightspeed computation of optimal transport. In: *Advances in Neural Information Processing Systems, Lake Tahoe, Nevada*. pp. 2292–2300.

[btac243-B9] Gibbs R.A. et al (2003) *The International Hapmap Project, United Kingdom*.

[btac243-B10] Goudet J. et al (2018) How to estimate kinship. Mol. Ecol., 27, 4121–4135. 30107060 10.1111/mec.14833 PMC6220858

[btac243-B11] Guo F. et al (2006) Fingerprinting relational databases. In: *Proceedings of the 2006 ACM Symposium on Applied Computing, Dijon, France*. pp. 487–492.

[btac243-B12] Halimi A. et al (2021) Privacy-preserving and efficient verification of the outcome in genome-wide association studies. *arXiv preprint arXiv:2101.08879.* 10.56553/popets-2022-0094 PMC9536480 36212774

[btac243-B13] Ji T. et al (2021a) The curse of correlations for robust fingerprinting of relational databases. In: *24th International Symposium on Research in Attacks, Intrusions and Defenses, RAID ’21, San Sebastian, Spain*. pp. 412–427. 10.1145/3471621.3471853 PMC10644290 37964942

[btac243-B14] Ji T. et al (2021b) Differentially-private fingerprinting of relational databases. *arXiv preprint arXiv:2109.02768.*

[btac243-B15] Johnson N.F. et al (2001) Information Hiding: Steganography and Watermarking-Attacks and Countermeasures: Steganography and Watermarking: Attacks and Countermeasures. Vol. 1. Springer Science & Business Media, Berlin/Heidelberg, Germany.

[btac243-B16] Lafaye J. et al (2008) Watermill: an optimized fingerprinting system for databases under constraints. IEEE Trans. Knowl. Data Eng., 20, 532–546.

[btac243-B17] Le K. et al (2021) On robust optimal transport: computational complexity and barycenter computation. In: Advances in Neural Information Processing Systems. Vol. 34.

[btac243-B18] Li J.Z. et al (2008) Worldwide human relationships inferred from genome-wide patterns of variation. Science, 319, 1100–1104. 18292342 10.1126/science.1153717

[btac243-B19] Li Y. et al (2003) Constructing a virtual primary key for fingerprinting relational data. In: *Proceedings of the 3rd ACM Workshop on Digital Rights Management, Washington DC.* pp. 133–141.

[btac243-B20] Li Y. et al (2005) Fingerprinting relational databases: schemes and specialties. IEEE Trans. Dependable Secure Comput., 2, 34–45. 10.1109/tdsc.2022.3191117 PMC10877201 38384377

[btac243-B21] Liu S. et al (2004) A block oriented fingerprinting scheme in relational database. In: *International Conference on Information Security and Cryptology, Seoul, Korea*. pp. 455–466. Springer.

[btac243-B22] McGee M.K. , Ross R. (2016) *4 Stolen Health Databases Reportedly for Sale on Dark Web*.

[btac243-B23] Mitchell A.A. et al (2004) Discrepancies in dbSNP confirmation rates and allele frequency distributions from varying genotyping error rates and patterns. Bioinformatics, 20, 1022–1032. 14764571 10.1093/bioinformatics/bth034

[btac243-B24] Naveed M. et al (2015) Privacy in the genomic era. ACM Comput. Surv., 48, 1–44. 10.1145/2767007 PMC4666540 26640318

[btac243-B25] Öksüz A.Ç. et al (2021) Privacy-preserving and robust watermarking on sequential genome data using belief propagation and local differential privacy. Bioinformatics, 37, 2668–2674. 33630065 10.1093/bioinformatics/btab128 PMC11025661

[btac243-B26] Park S.-J. et al (2013) Inference of kinship coefficients from Korean SNP genotyping data. BMB Rep., 46, 305–309. 23790973 10.5483/BMBRep.2013.46.6.177 PMC4133901

[btac243-B27] Pfitzmann B. , Waidner M. (1997) Asymmetric fingerprinting for larger collusions. In: *Proceedings of the 4th ACM Conference on Computer and Communications Security, Zurich, Switzerland.* pp. 151–160.

[btac243-B28] Sheskin D.J. (2003) Inferential statistical tests employed with two or more independent samples (and related measures of association/correlation). In: Sheskin,D.J. (ed.) Handbook of Parametric and Nonparametric Statistical Procedures. Chapman and Hall/CRC, New York, pp. 699–828.

[btac243-B29] Wang B. et al (2017) Efficient estimation of realized kinship from single nucleotide polymorphism genotypes. Genetics, 205, 1063–1078. 28100587 10.1534/genetics.116.197004 PMC5340323

[btac243-B30] Wheeler D.L. et al (2008) Database resources of the national center for biotechnology information. Nucleic Acids Res., 36, D13–D21. 18045790 10.1093/nar/gkm1000 PMC2238880

[btac243-B31] Yilmaz E. , Ayday E. (2020) Collusion-resilient probabilistic fingerprinting scheme for correlated data. *arXiv preprint arXiv:2001.09555.* 10.1007/978-3-031-37586-6_5 PMC12716443 41426237

